# The Long-Term Impact of Preterm Birth on Metabolic Bone Profile and Bone Mineral Density in Childhood

**DOI:** 10.3390/metabo15070463

**Published:** 2025-07-08

**Authors:** Panagiota Markopoulou, Artemis Doulgeraki, Arsinoi Koutroumpa, Georgios Polyzois, Helen Athanasopoulou, Christina Kanaka-Gantenbein, Tania Siahanidou

**Affiliations:** 1 Neonatology Unit, First Department of Pediatrics, Medical School, National and Kapodistrian University of Athens, “Aghia Sophia” Children’s Hospital, 11527 Athens, Greece; pmarkopoulou@med.uoa.gr (P.M.); ckanaka@med.uoa.gr (C.K.-G.); 2 Department of Bone and Mineral Metabolism, Institute of Child Health, 11527 Athens, Greece; doulgeraki@ich.gr (A.D.); giorgospolizois@gmail.com (G.P.); elathanasopoulou1@gmail.com (H.A.); 3 Second Neonatal Intensive Care Unit, “Aghia Sophia” Children’s Hospital, 11527 Athens, Greece; koutroumpaa@med.uoa.gr

**Keywords:** prematurity, bone mineral density, bone formation, bone resorption, long-term bone health

## Abstract

**Background/Objectives**: Recent data on long-term consequences of prematurity on bone health are conflicting. The aim of this study was to assess the metabolic bone profile and bone mineral density (BMD) in prepubertal children born prematurely and to examine possible associations between bone health parameters and perinatal morbidity factors. **Methods**: This cross-sectional observational study included 144 children of mean (SD) age 10.9 (1.6) years: 49 children born very preterm (≤32 gestational weeks), 37 moderate-to-late preterm (32^+1^ to 36^+6^ gestational weeks), and 58 born at term (controls). Serum levels of calcium/Ca, phosphorus/P, alkaline phosphatase/ALP, 25-hydroxyvitamin D/25(OH)D, bone formation markers (osteocalcin/OC, procollagen type I C-terminal propeptide/PICP, and insulin growth factor-1/IGF-1), and bone resorption markers (serum tartrate-resistant acid phosphatase 5b/bone TRAP5band urinary calcium-to-creatinine ratio) were measured. Total-body and lumbar-spine BMD and BMD Z-scores were calculated using dual-energy X-ray absorptiometry/DXA. **Results**: Children born very preterm showed significantly higher ALP, OC, PICP, and bone TRAP5b levels compared to controls, as well as compared to children born moderate-to-late preterm. Total-body and lumbar-spine BMD Z-scores were significantly lower in the very preterm-born group compared to controls. Gestational diabetes, preeclampsia, and bronchopulmonary dysplasia were associated with lower total-body BMD in the very preterm-born population. **Conclusions**: Preterm birth is associated with impaired metabolic bone profile and lower total-body and lumbar-spine BMD in childhood. Moderate-to-late preterm-born children exhibit altered metabolic bone parameters compared to very preterm-born children. Further research in children might allow better insight into the long-term impact of preterm birth on bone health.

## 1. Introduction

Preterm birth remains the leading cause for 75% of neonatal mortality and over half of long-term morbidity, as it is linked to severe physical, neurodevelopmental, and socioeconomic consequences, as well as to early onset of chronic diseases [1,2,3]. Metabolic bone disease of prematurity (MBDP), a complication of preterm birth, is characterized by inadequate mineralization of the preterm-born infant skeleton [4]. Specifically, MBDP is presented in 23% of neonates with very low birth weight (<1500 g) and in more than half of neonates with extremely low birth weight (<1000 g) [5,6,7], while the gestational age has been found to be inversely correlated to the risk of MBDP [8,9].

The final trimester of pregnancy is a critical period for the fetal skeletal development and mineralization; preterm birth interrupts the transplacental transport of calcium and phosphate, thus resulting in reduced bone mineralization in infants born prematurely [5,10]. Prolonged immobilization of preterm-born infants also affects bone growth, as it restricts spontaneous movements ex utero and reduces mechanical stimulation [8,11]. Furthermore, parenteral nutrition and inadequate enteral feeding significantly limit the mineral content provided to neonates born prematurely, while several prematurity-related complications, such as preeclampsia, bronchopulmonary dysplasia (BPD), neonatal sepsis, and administration of corticosteroids and/or diuretics, may also have adverse effects on skeletal development [10,12,13,14].

However, data on the long-term impact of preterm birth on skeletal health are still conflicting. Several studies have reported that young adults who were born prematurely, especially those with birth weight less than 1500 g, as well as those born <29 gestational weeks, have reduced areal bone mineral density (aBMD) of the lumbar spine [15,16,17], the femoral neck [15,16,17,18,19,20], and the whole body [15,21] compared to peers born full-term; the above findings are in concordance with similar studies conducted in preterm-born children and adolescents [17,22,23,24,25,26]. Moreover, prematurity has been associated with impaired bone strength index (BSI) and reduced cross-sectional bone dimensions adjusted to the body size in young adulthood [25]. Recent data have also demonstrated that adults who were born with very low birth weight (≤1500 g), or extremely preterm (≤28 gestational weeks), depict increased prevalence of osteopenia or osteoporosis, implying higher risk for future fractures [15,26]. On the contrary, other studies have shown that prematurity has no effect on bone health and aBMD in children, adolescents, or adults in the long term [27,28,29,30,31,32,33,34,35,36]. Furthermore, regarding bone formation and resorption markers, higher osteocalcin (OC) levels have been found in both children and adults born prematurely compared to their full-term peers [21,30,33,37]. The above elevation may reflect compensatory but ineffective bone remodeling, or high bone turnover, potentially contributing to reduced BMD in the preterm-born population [21,30,33,37]. However, other studies failed to identify any significant differences in bone formation and resorption markers between children or adults born prematurely and individuals born full-term [17].

It is noteworthy that studies linking prematurity to adverse long-term consequences on bone structure refer to the entire population of preterm-born individuals, including those born moderate-to-late preterm (32^+1^ to 36^+6^ gestational weeks) [14,38]. However, the available data specifically on moderate-to-late preterm individuals remain limited, and it has not yet been clarified whether this subgroup of preterm-born children differs in terms of skeletal growth and mineralization, as well as in the risk of osteopenia and/or osteoporosis, compared to either children born very prematurely (≤32 gestational weeks) or children born at term.

The aim of this study was to evaluate the metabolic bone profile and bone mineral density (BMD) of school-aged children born very preterm (≤32 gestational weeks) in comparison to children born at term and children born moderate-to-late preterm (32^+1^ to 36^+6^ gestational weeks), as well as to investigate potential associations between bone health parameters and perinatal morbidity-related factors within the preterm-born population.

## 2. Materials and Methods

### 2.1. Study Design and Participants

A cross-sectional observational study was performed at “Aghia Sofia” Children’s Hospital (Athens, Greece) in collaboration with the Institute of Child Health (Athens, Greece). A total of 144 school-aged children (mean age 10.9 ± 1.6 years) were included; of them, 86 children (39 males and 47 females) were born prematurely. The preterm-born group was further divided into two groups; group A consisted of 49 children born very preterm (≤32 gestational weeks), whereas group B consisted of 37 children who were born moderate-to-late preterm (32^+1^ to 36^+6^ gestational weeks). Forty-three of the participants born very preterm (Group A) and twenty-six of those born moderate-to-late preterm (Group B) had birth weight appropriate for gestational age (AGA), while three participants in Group A and ten participants in Group B were small for gestational age (SGA). AGA was defined as a birth weight between the 10th and 90th percentiles for gestational age, while SGA was defined as a birth weight below 10th percentile for gestational age [39]. The control group consisted of 58 healthy children (32 males and 26 females) born at term (37–42 gestational weeks); of them, 47 were born AGA, while 8 were born SGA.

All participants had been hospitalized at birth in the Neonatal Unit of the First Department of Pediatrics of National & Kapodistrian University of Athens, or in the Second Neonatal Intensive Care Unit, at “Aghia Sofia” Children’s Hospital (Athens, Greece) between 2007 and 2011, and they were randomly traced, by simple random sampling technique, from lists from the neonatal follow-up clinic, which provides regular follow-up to all infants admitted. Exclusion criteria were personal and/or family history of bone disease, history of fracture within the last three months, congenital malformations, chromosomal anomalies, active disease, metabolic or endocrine disease, supplementation with calcium and/or vitamin D in the last three months, and/or medications that may influence bone metabolism (e.g., systemic corticosteroids). The skeletal workup of the study population was performed at the Department of Bone and Mineral Metabolism of the Institute of Child Health and comprised bone mineral density assessment and evaluation of bone formation and bone resorption markers (bone turnover assessment). The study protocol was approved by the Research and Ethics Committee of “Aghia Sofia” Children’s Hospital, Athens, Greece, corresponding to the principles outlined in the Declaration of Helsinki. Written informed consent was obtained from parents and children.

### 2.2. Perinatal and Neonatal Data

Medical and family history was recorded for all participants, while perinatal and neonatal data were extracted from hospital medical records. Pregnancy complications were also documented; maternal gestational diabetes was defined as any degree of glucose intolerance with onset or first recognition during pregnancy [40]; gestational hypertension was defined as systolic blood pressure ≥140 mmHg and/or diastolic blood pressure ≥90 mmHg at ≥20 weeks of gestation in the absence of proteinuria or new signs of end-organ dysfunction [41]; and preeclampsia was defined as systolic blood pressure ≥140 mmHg and/or diastolic blood pressure ≥90 mmHg at ≥20 weeks of gestation in the presence of proteinuria or new signs of end-organ dysfunction [41]. Details regarding the neonatal unit hospitalization period and neonatal comorbidities were also recorded, including duration of parenteral nutrition and breast milk feeding, mechanical ventilation, respiratory distress syndrome (RDS), intraventricular hemorrhage (IVH), retinopathy of prematurity (ROP), patent ductus arteriosus (PDA), necrotizing enterocolitis (NEC), and BPD; BPD was defined as supplemental oxygen requirement at 36 weeks of postmenstrual age [42].

### 2.3. Clinical Assessment

The study participants attended the outpatient clinic in the morning, after a 12h overnight fast. Standing height was measured to the nearest 0.1 cm, while barefoot, using a Harpenden stadiometer (London, UK), and weight to the nearest 0.1 kg, via a scale (Seca 712, Birmingham, UK), with subjects wearing light clothing. Waist and hip circumference were also measured to the nearest 0.1 cm over an unclothed abdomen and at minimal respiration, with the use of an inextensible anthropometric tape (Seca 201, Birmingham, UK). The waist-to-hip ratio (WHR) and the BMI (weight in kilograms divided by height in meters squared) were calculated. Weight, height, and BMI were converted to age- and gender-specific Z-scores according to Centers for Disease Control and Prevention (CDC) growth reference charts [43]. All anthropometric measurements were performed twice; the mean was used for analysis. Pubertal stage was assessed using the Tanner and Whitehouse method [44]; stages 1 and 2 were classified as prepubertal, while stages 3, 4, and 5 were classified as pubertal.

History of fractures (number, location, and mechanism, as well as age at the time of the fracture) was also collected at the study visit, while participants provided information about estimated daily calcium intake (adequate intake reported as three to four portions of dairy per day; inadequate intake reported as zero to two portions of dairy per day [45]) and their physical activity (reported as hours of school and extracurricular physical activity per week) during the past 12 months. All participants belonged to a similar favorable socioeconomic background, which allowed them access to healthy nutrition for skeletal development and opportunities for regular exercise.

### 2.4. Metabolic Bone Profile

On the day of clinical assessment and total-body dual-energy X-ray absorptiometry (DXA) scan performance, basic bone profile was evaluated in all participants; fasting serum levels of calcium (Ca), phosphorus (P), alkaline phosphatase (ALP), and 25-hydroxyvitamin D [25(OH)D] were measured. Furthermore, fasting serum and second morning void urine samples were collected to evaluate bone formation and bone resorption markers (bone turnover) in the study population. The following bone formation markers were measured in serum: osteocalcin (OC) (Quidel corporation, Metra Biosystems, San Diego, CA, USA), procollagen type I C-terminal propeptide (PICP) (Quidel corporation, Metra Biosystems, San Diego, CA, USA), and insulin growth factor-1 (IGF-1) (Immunodiagnostic Systems Ltd., Boldon, Tyne & Wear, UK). For bone resorption, serum tartrate-resistant acid phosphatase 5b (bone TRAP5b) (Immunodiagnostic Systems Ltd., Boldon, Tyne & Wear, UK) and urinary calcium/creatinine ratio (uCa/uCr) were determined. The bone profile of the participants was evaluated by enzyme-linked immunoassay (ELISA), except for uCa/uCr, which was measured by atomic absorption (Shimadzu AA-6800 Spectrophotometer, Shimadzu, Tokyo, Japan).

### 2.5. Dual-Energy X-Ray Absorptiometry

A total-body dual-energy X-ray absorptiometry (DXA scan, Lunar Prodigy; GE Medical System, Slough, UK) and the accompanying pediatric software were used to determine bone mineral content (BMC, g), reflecting bone mass, lean tissue mass (LTM, g), total body fat mass (FM, g), and body fat percentage (BF, %). Lean tissue mass was calculated using the formula LTM (g) = FFM (g) − BMC (g), where FFM = fat free mass. Fat mass was derived from the equation FM (g) = Weight (g) × BF (%). Furthermore, total-body-less-head (TBLH) and lumbar-spine (L1–L4) bone mineral density (aBMD) (g/cm^2^) and the respective age- and gender-specific Z-scores were calculated by the incorporated software (enCore 2008, Lunar Prodigy, GE Medical Systems, V.17) using data for pediatric Mediterranean reference population [46]. All patients wore light, metal-free clothing. Patients with height <10th percentile or >90th percentile had their BMD Z-scores adjusted accordingly, using Ht–age, in accordance with pediatric guidelines for DXA interpretation [47]. All scans were performed by the same qualified technician. A thorough calibration procedure was completed daily with an appropriate phantom following the manufacturer’s protocol. Precision was expressed automatically with a coefficient of variation, which was 1.5% for lumbar-spine BMD and 1.1% for total-body BMD.

According to the official statement of the International Society for Clinical Densitometry (ISCD), the cut-off value for low aBMD Z-score is ≤−2.0, and defined as “low aBMD for chronological age” [48]. According to previous studies [14,18] and in order to identify minor bone dysfunction prior to the onset of osteopenia and/or osteoporosis in our study population, low TBLH and lumbar-spine (L1–L4) aBMD were defined as a subcranial total-body and lumbar-spine (L1–L4) aBMD Z-score ≤ −1.0 SD, respectively.

### 2.6. Statistical Analysis

Statistical analysis was performed using SPSS version 28.0 (SPSS Inc, Chicago, IL, USA). All growth parameters and DXA scan absolute values were converted to Z-scores, to permit comparisons between different age groups. The Kolmogorov–Smirnov and Shapiro–Wilk procedures were applied for testing normality of distribution. In parameters with normal distribution, results are given as mean ± SD; for parameters not normally distributed, results are expressed as medians and range (25th–75th percentile). Multiple group comparisons were performed using analysis of variance (ANOVA) or Kruskal–Wallis test (H test), as appropriate, for continuous variables, and chi-square test for categorical variables. Where significant differences were identified in multiple group comparisons, posthoc pairwise analyses were conducted using independent *t*-tests or Mann–Whitney U tests for continuous variables, and chi-square tests for categorical variables, as appropriate. Bonferroni correction was applied to adjust for multiple comparisons in all posthoc comparisons. In the tables, *p*-values from ANOVA and Kruskal–Wallis tests (H tests) are reported, whereas Bonferroni-adjusted *p*-values from pairwise comparisons are provided in the main text and in the Supplementary Materials’ tables. Linear correlations between variables of interest were calculated using Pearson’s or Spearman’s correlation coefficient for parameters with normal or skewed distribution, respectively. Multiple linear regression analysis was further performed to investigate independent associations of prematurity and bone densitometric parameters. Statistical significance was accepted if the null hypothesis could be rejected at *p*-value ≤ 0.05.

## 3. Results

### 3.1. Perinatal and Neonatal Characteristics

The perinatal and neonatal characteristics of our study population are shown in Table 1. Mean ± SD gestational age and birth weight of children born very preterm were 29.0 ± 2.2 gestational weeks and 1254.6 ± 353.8 g, respectively. Compared to controls, maternal preeclampsia, maternal smoking during pregnancy, and cesarean delivery were more common in the very preterm-born group (*p* = 0.01, *p* = 0.01, and *p* < 0.001, respectively), as well as in the moderate-to-late preterm-born group (*p* = 0.03, *p* = 0.002, and *p* < 0.001, respectively) (Table 1 and Table S1). Furthermore, almost 60% of children born very preterm and 47% of children born moderate-to-late preterm were exposed to antenatal corticosteroids, and 71% and 23% of them, respectively, received surfactant therapy as neonates (Table 1).

### 3.2. Anthropometrics and Metabolic Bone Profile

Children born very preterm presented with higher WHR (*p* = 0.001) compared to the controls (Table 2 and Table S2). No significant differences were found between children born very preterm and controls regarding current values of body weight, height, and BMI, as well as their Z-scores, waist circumference, and hip circumference (Table 2). Among children born prematurely, children born very preterm presented with higher BMI (*p* = 0.03), waist circumference (*p* = 0.001), and WHR (*p* = 0.002) compared to children born moderate-to-late preterm (Table 2 and Table S2). Fracture history and daily calcium intake did not differ significantly between groups; however, children born very preterm presented with reduced physical activity over the past 12 months compared to controls, as well as compared to children born moderate-to-late preterm (*p* = 0.005 and *p* = 0.002, respectively) (Table 2 and Table S2).

Regarding basic bone profile, children born very preterm showed significantly higher levels of ALP compared to children born at term (*p* = 0.008), as well as compared to children born moderate-to-late preterm (*p* < 0.001) (Table 2 and Table S2). No significant differences were found between very preterm-born children and either controls or moderate-to-late preterm children regarding serum calcium, phosphorus, and 25(OH)D levels (Table 2). Regarding the bone-formation markers examined, OC and PICP levels were significantly higher in children born very prematurely compared to children born at term (*p* = 0.04 and *p* = 0.009, respectively), as well as compared to children born moderate-to-late preterm (*p* < 0.001 and *p* = 0.001, respectively); IGF-1 levels in serum did not differ significantly between groups (Table 2 and Table S2). Regarding bone resorption markers, children born very prematurely presented with higher bone TRAP5b levels compared to controls (*p* = 0.01), as well as compared to children born moderate-to-late preterm (*p* < 0.001); no significant differences were found between both groups of preterm-born children and controls regarding uCa/uCr (Table 2 and Table S2).

In the total study population, levels of ALP were negatively correlated with gestational age (r_s_ = −0.28, *p* = 0.001) and birth weight (r_s_ = −0.19, *p* = 0.02). In the total preterm-born population, ALP, PICP, OC, and bone TRAP5b levels correlated negatively with gestational age (r_s_ = −0.43, *p* < 0.001; r_s_ = −0.41, *p* = 0.01; r_s_ = −0.60, *p* < 0.001; and r_S_ = −0.63, *p* < 0.001, respectively) and birth weight (r_s_ = −0.23, *p* = 0.02; r_s_ = −0.38, *p* = 0.01; r_s_ = −0.46, *p* = 0.003; and r_s_ = −0.37, *p* = 0.02, respectively).

### 3.3. Body Composition and Bone Densitometric Findings

Total-body-less-head BMD and BMD Z-scores were found significantly lower in the very preterm-born group compared to controls (*p* = 0.03 and *p* = 0.005, respectively) (Table 3 and Table S3); total-body BMC was significantly lower in the very preterm-born group compared to controls after correction for height at the time of measurement (*p* = 0.01). Furthermore, BMC, aBMD, and aBMD Z-score of the lumbar spine were also significantly lower in children born very preterm compared to controls (*p* = 0.05, *p* = 0.002 and *p* < 0.001, respectively) (Table 3 and Table S3); BMC of the lumbar spine remained significantly lower in the very preterm-born group compared to controls after correction for height at the time of measurement (*p* = 0.004). No significant differences were found between very preterm-born children and controls regarding LTM and FM, while no significant differences were depicted between children born very prematurely and children born moderate-to-late preterm regarding body composition and bone densitometric findings (Table 3).

In the total study population, gestational age correlated positively with TBLH BMD Z-score (r_s_ = 0.19, *p* = 0.03), as well as with aBMD and aBMD Z-score of the lumbar spine (r_s_ = 0.18, *p* = 0.04 and r_s_ = 0.25, *p* = 0.003, respectively). Moreover, birth weight correlated positively with TBLH BMD Z-score (r_s_ = 0.28, *p* = 0.001), as well as with lumbar-spine aBMD Z-score (r_s_ = 0.24, *p* = 0.005), in the total study population. In the total preterm-born population, TBLH BMD and aBMD of the lumbar spine correlated negatively with bone TRAP5b levels (r = −0.41, *p* = 0.01; and r = −0.61, *p* < 0.001, respectively).

After adjusting for height, puberty, and physical activity, being born very preterm remained an independent risk factor for lower TBLH BMC (β = −0.14, *p* = 0.01), BMD (β = −0.23, *p* = 0.02) and BMD Z-score (β = −0.28, *p* = 0.006), as well as for lower BMC (β = −0.22, *p* < 0.001), aBMD (β = −0.34, *p* < 0.001), and aBMD Z-score (β = −0.38, *p* < 0.001) of the lumbar spine. Moreover, in the total preterm-born population, after adjustment for height, puberty, and physical activity, PICP levels remained an independent risk factor for lower TBLH BMC (β = −0.19, *p* = 0.03), BMD (β = −0.29, *p* = 0.03), and BMD Z-score (β = −0.62, *p* = 0.01), while bone TRAP5b levels were recognized as an independent risk factor for lower BMC and aBMD of the lumbar spine (β = −0.18, *p* = 0.05; and β = −0.44, *p* = 0.01, respectively).

As expected, prematurity less than 32 weeks of gestation was associated with a significantly larger proportion of children with low TBLH BMD, as well as low aBMD of the lumbar spine; four very preterm-born children, compared to none in the control group, were found to have a TBLH BMD Z-score ≤ −1.0 SD (X^2^ = 4.92, *p* = 0.03), while eleven very preterm-born children, compared to four controls, were found to have a lumbar-spine aBMD Z-score ≤ −1.0 SD (X^2^ = 5.33, *p* = 0.02). Values of total-body and lumbar-spine BMD Z-score ≤ −2.5 were not observed in our study population; therefore, no systematic surveillance for vertebral fractures using lateral spine X-rays was conducted. Furthermore, being born very prematurely was associated with a higher risk for a low TBLH BMD Z-score (OR 4.88, 95% CI 1.47, 16.14, *p* = 0.006), as well as a low aBMD Z-score of the lumbar spine (OR 3.91, 95% CI 1.16, 13.20, *p* = 0.02) during childhood.

### 3.4. Correlations of Metabolic Bone Profile and Bone-Densitometric Findings with Perinatal and Neonatal Characteristics

In the very preterm-born group, children born to mothers with gestational diabetes presented with significantly lower LTM (22,685.9 ± 3435.4 g vs. 28,374.9 ± 5866.2 g, *p* = 0.02), TBLH BMC (1188.6 ± 175.3 g vs. 1445.6 ± 298.7 g, *p* = 0.03), TBLH BMD (0.78 ± 0.03 g/cm^2^ vs. 0.83 ± 0.08 g/cm^2^, *p* = 0.01), and BMC of the lumbar spine (24.6 ± 2.7 g vs. 29.2 ± 6.9 g, *p* = 0.01) compared to children born to non-diabetic mothers. Furthermore, very preterm-born children born to mothers with preeclampsia presented with significantly lower LTM (23,898.6 ± 4974.4 g vs. 28,151.5 ± 5881.7 g, *p* = 0.05), TBLH BMD (0.76 ± 0.09 g/cm^2^ vs. 0.83 ± 0.07 g/cm^2^, *p* = 0.04), and BMD Z-score (−0.27 ± 0.62 vs. 0.50 ± 0.94, *p* = 0.04) compared to very preterm-born children born to non-preeclamptic mothers.

In the very preterm-born population, the presence of BPD was associated with significantly lower TBLH BMD and BMD Z-score (0.79 ± 0.08 g/cm^2^ vs. 0.85 ± 0.07 g/cm^2^, *p* = 0.01 and 0.08 ± 0.98 vs. 0.70 ± 0.80, *p* = 0.02, respectively), as well as aBMD and aBMD Z-score of the lumbar spine (0.72 ± 0.09 g/cm^2^ vs. 0.79 ± 0.11 g/cm^2^, *p* = 0.03 and −0.66 ± 0.80 vs. −0.12 ± 0.87, *p* = 0.04, respectively). After adjustment for height and puberty, BPD remained an independent risk factor for lower TBLH BMC (β = −0.17, *p* = 0.03), TBLH BMD (β = −0.30, *p* = 0.004), TBLH BMD Z-score (β = −0.31, *p* = 0.03), BMC (β = −0.19, *p* = 0.04), and aBMD (β = −0.26, *p* = 0.05) of the lumbar spine in the very preterm-born population. Metabolic bone profile and bone densitometric parameters studied were not found to be associated with other perinatal morbidity factors or the duration of parenteral nutrition and/or breast milk feeding in the very preterm-born population.

### 3.5. Characteristics of Preterm-Born Children with Low BMD

A subgroup analysis for the very preterm-born group was performed comparing those with either total-body BMD Z-score ≤ −1.0 SD or lumbar-spine aBMD Z-score ≤ −1.0 SD to those with normal BMD. We did not identify any perinatal or neonatal factors associated with TBLH BMD Z-score ≤ −1.0 SD or lumbar-spine aBMD Z-score ≤ −1.0 SD in children born very prematurely (Table 4). However, very preterm-born children with low BMD presented with significantly lower weight and weight Z-score (*p* = 0.01 and *p* < 0.001, respectively), BMI, and BMI Z-score (*p* = 0.001 and *p* = 0.002, respectively), as well as waist circumference (*p* = 0.002), hip circumference (*p* = 0.004), and WHR (*p* = 0.05), compared to very preterm-born children with normal BMD (Table 4). Furthermore, low BMD was associated with inadequate daily calcium intake in the very preterm-born population (β = 0.63, *p* < 0.001). Regarding metabolic bone profile, very preterm-born children with low BMD presented with significantly lower IGF-1 levels compared to very preterm-born children with normal BMD (*p* = 0.04) (Table 4). No significant differences were found regarding fracture history or physical activity over the past 12 months, or any other metabolic bone profile parameter examined between children with low BMD and children with normal BMD in the very preterm-born group.

### 3.6. Comparisons of Anthropometrics, Metabolic Bone Profile Parameters, Body Composition, and Bone Densitometric Findings Between Children Born Moderate-to-Late Preterm and Controls

No significant differences were found regarding body weight, height, and BMI, as well as for Z-scores, waist circumference, hip circumference, and WHR, between children born moderate-to-late preterm and controls. Regarding metabolic bone profile, OC and PICP, as well as bone TRAP5b levels, were significantly lower in children born moderate-to-late preterm compared to children born at term (*p* = 0.002, *p* = 0.05, and *p* = 0.003, respectively) (Table 2 and Table S2). Furthermore, TBLH BMD Z-scores and aBMD Z-scores of the lumbar spine were significantly lower in the moderate-to-late preterm-born population compared to controls (*p* = 0.002 and *p* = 0.05, respectively) (Table 3 and Table S3). No significant differences were found regarding other metabolic bone profile parameters, body composition, and bone densitometric findings between children born moderate-to-late preterm and controls.

## 4. Discussion

In this study, children born very prematurely (≤32 gestational weeks) exhibited an impaired metabolic bone profile with significantly higher levels of alkaline phosphatase (ALP), osteocalcin (OC), procollagen type I C-terminal propeptide (PICP), and serum tartrate-resistant acid phosphatase 5b (bone TRAP5b) compared to both children born moderate-to-late preterm and children born at term. Furthermore, the very preterm-born group exhibited an impaired profile in bone densitometric parameters; total-body-less-head BMC, BMD, and BMD Z-score, as well as BMC, aBMD, and aBMD Z-score of the lumbar spine, were significantly lower in children born very prematurely compared to controls. No significant differences were observed between children born very prematurely and children born moderate-to-late preterm in terms of body composition and bone densitometric findings. After adjustment for height, puberty, and physical activity, very preterm birth was identified as an independent risk factor for lower total-body-less-head BMC, BMD, and BMD Z-score, as well as lower BMC, aBMD, and aBMD Z-score of the lumbar spine. This indicates that prematurity has a negative effect on bone health that is already evident during childhood. Regarding the long-term consequences of prematurity-related perinatal morbidity on skeletal status, this study is the first to demonstrate the negative impact of gestational diabetes, preeclampsia, and BPD on bone densitometric parameters in children born very prematurely.

Worldwide, 1 in 10 infants is born prematurely (<37 weeks of gestation), and the global incidence rate has remained significant during the past decade; current data indicate that 152 million neonates were born before 37 gestational weeks between 2010 and 2020, while the global rate of preterm birth was 9.9% in 2020 [1,2,3]. Furthermore, survival rates of preterm-born infants have dramatically improved, with 50% of neonates born at 24 gestational weeks and 90% of those born at 28 gestational weeks in high-income countries surviving beyond the neonatal period [49]. Osteoporosis is concerned as a major public health concern in the developed world; it has a significant impact on mortality and quality of life, and leads to increased healthcare costs [50]. Several studies have reported that osteoporosis reflects an imbalance in bone remodeling, characterized by bone resorption exceeding bone formation, and is associated with impaired skeletal microstructure and increased fracture risk [51]. DXA is the most frequently used technique for assessing bone mineralization in childhood and adolescence [52,53]. Reduced BMD measurements by DXA are recognized as significant determinants of osteoporosis and fracture risk in adulthood [51,54].

During the neonatal period, prematurity is associated with impaired skeletal development and deficient bone mineralization, primarily due to severe calcium and phosphorus deficiency. Preterm birth severely reduces the active transplacental transport of minerals during the third trimester of pregnancy, where up to 80% of fetal bone mineral accumulation occurs [5,10,55]. Additionally, minimal enteral feeding, parenteral nutrition, and poor fat absorption play crucial roles in impairing bone mineralization in preterm infants [23]. Furthermore, preterm-born infants have a limited range of movements exutero [8,11], and medications such as diuretics and corticosteroids, along with perinatal morbidity, may further adversely affect bone microarchitecture [55]. Severe under-mineralization during the critical period of neonatal age may significantly influence the attainment of peak bone mass and increase the fracturerisk in preterm-born individuals during childhood and early adolescence, as such measures are key predictors for osteoporosis in adulthood.

Data on the long-term impact of preterm birth on skeletal health and peak BMD remain conflicting. Starting in infancy, preterm-born infants have been reported to have lower BMC and BMD at term-corrected age [56,57,58,59]. Only a limited number of studies have investigated the potential impact of preterm birth on bone densitometric parameters up until childhood. Our findings are in accordance with previous research showing that children born prematurely exhibited lower total-body BMC, as well as lower aBMD at the lumbar spine, lower hip, and femoral neck [14,22,23,24,57,59,60,61]. On the contrary, several studies have failed to demonstrate any significant differences between preterm-born children and children born at term [27,28,29,30,32,33,35]. A possible explanation may lie in the timing of follow-up, as previous cross-sectional studies have shown that infants born prematurely can exhibit catch-up in bone mass by two years of age [62,63]. However, more recent evidence suggests that normal bone mineralization after preterm birth may not occur until late childhood or adolescence [22,37,59,64,65]. Regarding the impact of prematurity on skeletal health in adulthood, recent studies have shown that adults born prematurely present with lower aBMD compared to controls [15,16,18,19,21,66], while others have failed to demonstrate any differences between preterm-born adults and those born at term [34,35,36,67].

Few studies have investigated the potential impact of perinatal morbidity factors on bone health in preterm-born individuals, and most have failed to identify any perinatal factors linked to lower BMC and/or BMD in this population [18]. Among others, NEC, IVH, and ROP may adversely affect bone health in children born prematurely [14,68,69]. Specifically, preterm children with a history of NEC exhibited lower total-body BMC, total-body BMD Z-score, and aBMD Z-score of the lumbar spine compared to those without NEC history [68]. Additionally, ROP and IVH have been associated with lower total BMC, while IVH has also been linked to reduced aBMD and aBMD Z-score of the lumbar spine in preterm-born children [14].

This study is the first to demonstrate the negative impact of gestational diabetes on total-body BMC and BMD, as well as lumbar-spine BMC, and of maternal preeclampsia on total-body BMD and BMD Z-score in children born prematurely. Both gestational diabetes and preeclampsia are recognized as important risk factors for MBPD in infancy [6,70,71]. Miettola et al. were the first to underline that the long-term skeletal health outcomes of preterm-born infants exposed to maternal preeclampsia differ significantly from those of other preterm-born individuals in adulthood [12]. Furthermore, in this study, having a history of BPD was associated with lower total-body BMD and BMD Z-score, as well as lower aBMD and aBMD Z-score of the lumbar spine in the very preterm-born population. Moreover, BPD was identified as an independent risk factor for reduced total-body BMC and BMD, along with lower BMC and aBMD of the lumbar spine, in children born very prematurely. Recent data indicate that preterm infants with BPD have impaired bone growth and an increased risk of MBDP [13,72]. To our knowledge, this is the first study to investigate the potential long-term impact of BPD on bone health in prematurely born individuals during childhood. Given the observed associations of gestational diabetes, preeclampsia, and BPD with impaired skeletal outcomes in children born prematurely, individualized bone-monitoring strategies and follow-up care tailored to perinatal complications may be warranted to mitigate long-term skeletal risks in the preterm-born population. Consistent with previous studies, our findings of disrupted bone metabolic profiles and impaired BMD in preterm-born children support the implementation of structured bone-monitoring pathways, especially in those with multiple perinatal morbidity factors [55,73].

An impaired metabolic bone profile in children born prematurely has been reported in a limited number of studies. The inadequate extracellular mineral concentration in preterm-born fetuses has a negative impact on the activity of osteoblasts and osteoclasts, which play crucial roles in bone formation and resorption, respectively [74]. Previous research has shown that OC levels are significantly elevated in children born prematurely compared to controls [33,37], in concordance with our findings. Elevated OC levels have also been observed in infants born prematurely at estimated full-term gestation [75], as well as in preterm-born adults compared to adults who were born at term [21]. OC is a 49 kDa protein produced by osteoblasts in the bone matrix, and it is recognized as a predictor of impaired bone mass in infants born prematurely [76,77]. Regarding other bone formation markers, PICP quantitatively reflects the synthesis of type I collagen and is mainly produced by proliferating osteoblasts [78]. Limited studies have reported increased PICP levels in infants born prematurely compared to controls [79]. To our knowledge, this is the first study to evaluate both OC and PICP levels in children born very prematurely compared to those born full-term. The observed alterations in the metabolic bone profile may reflect an upregulation of bone formation in very preterm-born children as a compensatory mechanism to maintain adequate bone mass, as well as a response to mineral insufficiency during the critical early period of skeletal development, which persists into childhood [37].

Regarding bone resorption, this study demonstrates, for the first time, significant elevation of bone TRAP5b levels in children born very prematurely compared to controls. Bone TRAP5b is an isoform of tartrate-resistant acid phosphatase (TRAP) enzyme and plays an important role as a marker of osteoclast activity and bone resorption [80]. A previous study in neonates demonstrated a positive correlation between bone TRAP5b activity and both gestational age and birth weight, suggesting that bone resorption is progressively activated during fetal growth [81]. However, the association between bone TRAP5b levels and prematurity has not yet been studied later in childhood. Similar to our findings, previous studies have reported high bone turnover in infants born prematurely [75,76,77], a finding which may underlie the pathophysiology of MBDP. Furthermore, ALP is considered an important surrogate biomarker of MBDP and increased bone turnover in infants born prematurely [10,82]; however, few studies have investigated ALP levels in preterm-born individuals beyond the neonatal period [14].

Limited data exist on individuals born moderate-to-late preterm, particularly regarding whether this group differs from those born very preterm (≤32 weeks of gestational age) in terms of bone growth and mineralization, and future risk of osteopenia or osteoporosis in childhood or adulthood. Concerning the severity of prematurity, previous studies have shown that impaired fetal growth plays a crucial role in bone densitometric parameters; thus, preterm SGA individuals may exhibit reduced BMC and aBMD compared to preterm AGA individuals in adulthood [35]. However, in a large cohort of adults born prematurely, gestational age was not found to be a key determinant of BMD variance [34]. In our study population, very preterm-born children showed no significant differences in bone densitometric parameters compared to moderate-to-late preterm children. To our knowledge, this is the first study to examine body composition and bone densitometric parameters between children born very prematurely and those born moderate-to-late preterm.

Novel findings of our study are also the increased levels of OC, PICP, and bone TRAP5b in children born very prematurely compared to those born moderate-to-late preterm. Elevated OC levels indicate increased bone turnover and remodeling activity, while increased PICP levels are a marker of enhanced collagen synthesis and can be indicative of upregulated extracellular matrix production [83,84]. These differences suggest impaired bone formation, altered collagen synthesis, and osteoblastic dysregulation in moderate-to-late preterm-born children compared to very preterm-born individuals upon childhood. Interestingly, since OC is involved in metabolic regulation, the reduced OC levels in moderate-to-late preterm-born children may be linked to impaired insulin sensitivity and glucose metabolism, potentially increasing their risk of insulin resistance [85]. Regarding the bone resorption markers examined in our study population, the decreased bone TRAP5b levels in moderate-to-late preterm-born children may indicate reduced osteoclastic activity and bone resorption. This could potentially explain the lack of significant differences in bone densitometric parameters between children born moderate-to-late preterm and those born very preterm.

Regarding the fracture rate, children born very prematurely had similar fracture incidence compared to controls, aligning with the findings of previous studies in preterm and low-birth-weight children [27,32]. This may reflect the significant advancements in neonatal intensive care and a generally more “bone-friendly” lifestyle after preterm birth [32]. Specifically, children and adolescents born very prematurely may have lower-risk behavior [86], as they tend to participate less in sports due to a higher burden of chronic diseases and reduced cardiorespiratory fitness [24,87,88]; physical activity is associated with increased fracture risk, although it improves BMD in childhood. In line with the above mentioned findings, our analysis showed that children born very prematurely had lower physical activity levels compared to both term-born controls and those born moderate-to-late preterm. Long-term follow-up of the above populations is needed in order to examine if the lower BMD observed in childhood among individuals born prematurely may influence the incidence of any fractures later in adulthood.

Nevertheless, regarding the potential influence of exercise, being born very preterm remained an independent risk factor for lower TBLH BMC, BMD, and BMD Z-score, as well as for reduced BMC, aBMD, and aBMD Z-score of the lumbar spine, even after adjusting for physical activity in our study population. Regular exercise during childhood and adolescence plays a crucial role in bone health, as it is associated with significant increases in BMC and BMD; it leads to 0.6–1.7% greater annual increase in bone accrual, with the prepubertal and peri-pubertal periods being the most responsive windows for skeletal adaptation [89]. Regular, high-impact physical activity during childhood and adolescence is a key strategy for improving bone health and against future osteoporosis risk [90]. In preterm-born infants, daily and brief (5–10min) passive range-of-motion exercises or physiotherapy interventions initiated during the neonatal period and maintained for 4–8 weeks have been associated with increased bone mineral content and bone strength [91,92]. However, while short-term benefits are evident, data on long-term effects of exercise on bone health in children or adolescents born prematurely are limited. Regular, age-appropriate physical activity throughout childhood and adolescence may be beneficial for preterm-born individuals, and informing parents and healthcare providers about the importance of exercise for bone health in preterm-born children is essential. Further research is needed for the development of targeted exercise recommendations and follow-up strategies aimed at optimizing skeletal outcomes in the preterm-born population.

Several studies have demonstrated that preterm-born children present with lower weight and height compared to term-born controls, which may negatively affect aBMD. In our study population, however, no significant differences were found in weight, height, or lean body mass between preterm-born children and controls. Notably, among very preterm-born children, those with low BMD exhibited lower weight and BMI compared to their peers with normal BMD. Previous research suggests that such anthropometric differences are mainly driven by preterm children born SGA, rather than those born AGA [35]; however, only 6% of our very preterm-born population was born SGA. Interestingly, in our analysis, preterm birth was recognized as an independent risk factor for lower total-body-less-head BMC, BMD, and BMD Z-score, as well as for decreased lumbar-spine BMC, aBMD, and aBMD Z-score, even after adjusting for body size where appropriate.

Since prematurity has been identified as an independent risk factor for lower total-body-less-head BMD and aBMD of the lumbar spine, and given that both very preterm and moderate-to-late preterm children in our study exhibited impaired metabolic bone profiles, the importance of long-term follow-up of bone health in individuals born preterm is emphasized. Compromised bone mineralization and altered bone metabolism during early life may have lasting effects on skeletal development and fracture risk throughout childhood, adolescence, and adulthood. While the DXA scan remains the gold standard for BMD assessment [93], non-invasive and radiation-free techniques, such as Radiofrequency Echographic Multi Spectrometry (REMS), may offer safe and repeatable bone health surveillance in preterm-born population during childhood and adolescence [94]. REMS is an emerging, non-ionizing, ultrasound-based technology for the assessment of BMD and bone quality at axial skeletal sites, including the lumbar spine and femoral neck [94]. Its key advantages include portability, absence of ionizing radiation, and suitability for use in populations where performance of DXA may be difficult or contraindicated, such as neonates or pregnant women [94]. However, further research is essential in order to investigate the potential role of REMS in the long-term follow-up of bone health in individuals born prematurely and to validate its clinical utility in this vulnerable population [71].

Our findings regarding the bone health of children born prematurely may also have important implications for maternal health recommendations before and during pregnancy. According to our findings, being born very preterm (≤32 gestational weeks) was recognized as an independent risk factor for lower total-body-less-head BMC, BMD, and BMD Z-score, as well as for lower BMC, aBMD, and aBMD Z-score of the lumbar spine. Therefore, maternal health recommendations aiming to prevent preterm birth, such as smoking cessation and nutritional optimization, including multiple micronutrient supplementation and balanced protein/energy intake, are crucial [95]. The observed associations between gestational diabetes and preeclampsia with lower total-body BMD in the very preterm-born population further highlight the importance of early and regular prenatal care, including screening for and managing these conditions. Optimal preconception management of diabetes mellitus and hypertension through lifestyle interventions, such as a well-balanced diet and appropriate weight control, is also crucial in improving overall maternal health. Furthermore, ensuring adequate maternal calcium intake; maintaining vitamin D sufficiency; and supporting a balanced diet rich in protein, phosphorus, and micronutrients are key recommendations to support fetal skeletal development, which may influence the future bone health of offspring [96].

An important strength of this study lies in the novelty of the results regarding the altered metabolic bone profile in children born very prematurely compared to those born at term. Additionally, this is the first study to investigate and compare metabolic bone parameters and bone densitometric findings between very preterm-born children and those born moderate-to-late preterm. Another novel outcome of this study is the identification of associations between prematurity-related perinatal morbidities and both metabolic bone profiles and bone densitometric parameters in children born very prematurely. In this study, a holistic approach to the evaluation of skeletal profile was taken, combining clinical, imaging (bone densitometry), and laboratory data (bone turnover markers), and also taking into account lifestyle parameters (diet and exercise), as well as fracture history. Furthermore, the present study has investigated both BMC and BMD of subcranial total body and lumbar spine, as well. To our knowledge, BMC and aBMD are the most accurate indicators of bone health in young children [97,98], and the total body less head and lumbar spine are recognized as the optimal sites for DXA measurements in childhood, reflecting both cortical and trabecular bone composition [69,99]. Despite significant advances in neonatal care, MBDP remains a significant complication of prematurity and may extend from childhood and into adulthood, with increased risk of impaired bone density and long-term skeletal complications. Further research is required to determine the optimal timing for follow-up of preterm-born populations and to assess the potential for catch-up in bone mass over time. The impact of preterm birth and prematurity-related perinatal morbidity on future bone health is still largely unknown, as onlya limited number of studies have investigated whether the bone profile disturbances of preterm-born infants persist in late childhood and until puberty; the above mentioned is one of the main strengths of this study.

This study also has a few limitations. Firstly, although we enrolled an adequate number of preterm-born children to ensure statistically significant results for the primary outcomes of metabolic bone profile and bone densitometric parameters, this sample size may not have been sufficient enough to uncover potential associations with nutritional interventions during the neonatal hospitalization. Secondly, the potential association between current total-body BMD and aBMD of the lumbar spine and a neonatal diagnosis of MBDP could not be assessed, as MBDP was not systematically documented in our records. Furthermore, the information on daily calcium intake and physical activity was based on the validity of self-report information from study participants and their parents.

## 5. Conclusions

In conclusion, very preterm birth (≤32 gestational weeks) is related to significantly higher levels of ALP, OC, PICP, and bone TRAP5b, as well as to lower total-body and lumbar-spine ΒMC, aBMD, and aBMD Z-score in childhood. Moderate-to-late preterm birth (32^+1^ to 36^+6^ gestational weeks) is associated with lower levels of ALP, OC, PICP, and bone TRAP5b compared to very preterm birth, which may suggest that BMD deficit in this subgroup is mainly driven by impaired osteoblastic activity and bone remodeling, rather than by increased bone resorption. Prematurity at ≤32 gestational weeks is recognized as an independent risk factor for lower total-body and lumbar-spine BMC, BMD, and BMD Z-score, indicating that the adverse effects of very preterm birth on bone health are already evident in childhood. Moreover, gestational diabetes, maternal preeclampsia, and BPD appear to exert an additional negative impact on bone densitometric parameters in this population. Further research focusing on the evaluation of the metabolic bone profile, including both bone formation and resorption biomarkers, along with bone densitometric parameters and their associations with prematurity-related comorbidities, is needed to provide a better insight into the long-term impact of preterm birth on bone health.

## Figures and Tables

**Table 1:** Perinatal and neonatal characteristics in preterm-born children and in controls.

Variable	Children Born Preterm (*n* = 86)		Controls (*n* = 58)	*p*-Value *
	Group A (*n* = 49)	Group B (*n* = 37)		
Age (years)	10.8 ± 1.2	11.0 ± 2.0	10.7 ± 1.5	0.40
Males (*n*)	24	15	32	0.42
Small for gestational age (SGA) [*n* (%)]	3 (6.1)	10 (27.0)	8 (13.8)	0.06
Maternal age at birth (years)	33.9 ± 4.8	32.8 ± 5.5	33.3 ± 4.7	0.76
Maternal gestational hypertension [*n* (%)]	1 (2.0)	1 (3.6)	3 (5.2)	0.41
Maternal preeclampsia [n (%)]	7 (14.3) ^†^	5 (13.9) ^#^	1 (1.7)	**0.05**
Maternal gestational diabetes [*n* (%)]	7 (14.3)	8 (22.2)	9 (15.5)	0.58
Maternal smoking during pregnancy [*n* (%)]	7 (14.3) ^†^	8 (22.2) ^†^	1 (1.7)	**0.007**
Antenatal corticosteroids [*n* (%)]	29 (59.2) ^‡§^	13 (46.4) ^‡^	4 (6.9)	**<0.001**
Cesarean delivery [*n* (%)]	44 (89.8) ^‡^	35 (94.6) ^‡^	27 (46.6)	**<0.001**
Gestational age (weeks)	29.0 ± 2.2 ^‡¶^	34.4 ± 1.4 ^‡^	38.9 ± 1.0	**<0.001**
Birth weight (g)	1254.6 ± 353.8 ^‡¶^	2011.1 ± 458.1 ^‡^	3241.8 ± 491.7	**<0.001**
RDS [*n* (%)]	40 (81.6) ^‡¶^	13 (37.1) ^‡^	0 (0)	**<0.001**
Surfactant therapy [*n* (%)]	35 (71.4) ^‡¶^	8 (22.9) ^‡^	0 (0)	**<0.001**
Mechanical ventilation [*n* (%)]	40 (81.6) ^‡¶^	9 (25.7) ^‡^	0 (0)	**<0.001**
Duration of mechanical ventilation (days)	12.0 (2.0–30.5) ^‡¶^	0 (0–2.0) ^‡^	0 (0)	**<0.001**
Duration of parenteral nutrition (days)	30.5 (15.8–44.0) ^‡§^	9 (2.0–32.5) ^#^	0 (0)	**0.02**
BPD [*n* (%)]	19 (38.8) ^‡¶^	0 (0)	0 (0)	**<0.001**
IVH [*n* (%)]	17 (34.7) ^‡¥^	4 (11.4) ^#^	0 (0)	**<0.001**
ROP [*n* (%)]	16 (32.7) ^‡¥^	4 (14.3) ^†^	0 (0)	**<0.001**
PDA [*n* (%)]	12 (24.5) ^‡¶^	0 (0)	0 (0)	**<0.001**
NEC [*n* (%)]	3 (6.1)	1 (2.9)	0 (0)	0.19

SGA, small for gestational age; RDS, respiratory distress syndrome; BPD, bronchopulmonary dysplasia; IVH, intraventricular hemorrhage; ROP, retinopathy of prematurity; PDA, patent ductus arteriosus; NEC, necrotizing enterocolitis. Group A: children born very preterm (≤32 gestational weeks). Group B: children born moderately or late preterm (32^+1^ to 36^+6^ gestational weeks). Statistical significance is defined by *p*-value less than or equal to 0.05 and statistically significant results are shown in bold type; * *p*-values from analysis of variance (ANOVA) or Kruskal–Wallis test (H test), as appropriate, for continuous variables and from chi-square test for categorical variables. For pairwise subgroup comparisons, ^#^ *p* ≤ 0.05, ^†^ *p* ≤ 0.01, and ^‡^ *p* ≤ 0.001 in comparison with controls; and ^§^ *p* ≤ 0.05, ^¥^ *p* ≤ 0.01, and ^¶^ *p* ≤ 0.001 in comparison with Group B, after Bonferroni correction. Exact Bonferroni-adjusted *p*-values from posthoc pairwise subgroup comparisons are mentioned in the main text and in Table S1 (Supplementary Materials).

**Table 2:** Anthropometric characteristics and metabolic bone profile in preterm-born children and in controls.

Variable	Children Born Preterm (*n* = 86)		Controls (*n* = 58)	*p*-Value *
	Group A (*n* = 49)	Group B (*n* = 37)		
Weight (kg)	41.6 ± 11.2	39.1 ± 10.7	39.9 ± 8.7	0.13
Weight Z-score	0.50 ± 0.99	0.33 ± 0.83	0.55 ± 0.81	0.29
Height (cm)	145.8 ± 9.9	145.5 ± 11.7	145.1 ± 10.1	0.57
Height Z-score	0.38 ± 0.93	0.32 ± 0.89	0.46 ± 0.91	0.79
BMI (kg/m^2^)	19.4 ± 3.9 ^§^	18.1 ± 3.0	18.8 ± 2.7	0.07
BMI Z-score	0.42 ± 1.27	0.23 ± 0.89	0.47 ± 0.98	0.36
Waist circumference (cm)	72.8 ± 10.6 ^¶^	68.7 ± 9.1	69.5 ± 8.0	**0.02**
Hip circumference (cm)	79.2 ± 9.7	79.7 ± 9.8	79.0 ± 7.8	0.63
WHR	0.92 ± 0.05 ^‡¥^	0.87 ± 0.05	0.88 ± 0.04	**<0.001**
Tanner stage 1–2 [*n* (%)]	34 (69.4)	25 (67.6)	42 (72.4)	0.06
Previous fracture history [*n* (%)]	1 (2.0)	0 (0)	5 (8.6)	0.31
Inadequate daily calcium intake [*n* (%)]	7 (14.3)	3 (8.1)	5 (8.6)	0.07
Physical activity (hours per week)	5.0 (4.0–7.0) ^†¥^	6.3 (5.0–9.8)	6.0 (4.0–8.0)	**0.006**
Calcium (mg/dL)	9.80 ± 0.21	9.71 ± 0.33	9.70 ± 0.28	0.08
Phosphorus (mg/dL)	4.46 ± 0.41	4.45 ± 0.40	4.55 ± 0.43	0.49
Alkaline phosphatase (IU/L)	274.49 ± 68.89 ^†¶^	213.67 ± 79.47	234.87 ± 80.73	**<0.001**
25(OH)D (ng/mL)	22.88 ± 9.03	27.18 ± 10.13	23.05 ± 10.54	0.17
Osteocalcin (OC) (ng/mL)	27.08 ± 5.24 ^#¶^	14.75 ± 9.3 ^†^	22.72 ± 9.11	**<0.001**
Procollagen type I C-terminal propeptide (PICP) (ng/mL)	475.69 ± 291.85 ^†¶^	234.72 ± 97.00 ^#^	302.34 ± 153.16	**<0.001**
Insulin growth factor-1 (IGF-1) (ng/mL)	308.67 ± 162.72	275.64 ± 115.94	256.47 ± 123.85	0.59
Serum tartrate-resistant acid phosphatase 5b (bone TRAP5b) (U/L)	7.91 ± 1.99 ^†¶^	4.80 ± 2.09 ^†^	6.45 ± 1.72	**<0.001**
Urinary calcium/creatinine (uCa/uCr)	0.26 (0.12–0.52)	0.23 (0.15–0.39)	0.23 (0.08–0.47)	0.47

BMI, body mass index; WHR, waist-to-hip ratio; 25(OH)D, 25-hydroxyvitamin D. Group A: children born very preterm (≤32 gestational weeks). Group B: children born moderately or late preterm (32^+1^ to 36^+6^ gestational weeks). Statistical significance is defined by *p*-value less than or equal to 0.05 and statistically significant results are shown in bold type; * *p*-values from analysis of variance (ANOVA) or Kruskal–Wallis test (H test), as appropriate, for continuous variables and from chi-square test for categorical variables. For pairwise subgroup comparisons, ^#^ *p* ≤ 0.05, ^†^ *p* ≤ 0.01, and ^‡^ *p* ≤ 0.001 in comparison with controls; and ^§^ *p* ≤ 0.05, ^¥^ *p* ≤ 0.01, and ^¶^ *p* ≤ 0.001 in comparison with Group B, after Bonferroni correction. Exact Bonferroni-adjusted *p*-values from posthoc pairwise subgroup comparisons are mentioned in the main text and in Table S2 (Supplementary Materials).

**Table 3:** Body composition and bone densitometric findings in preterm-born children and in controls.

Variable	Children Born Preterm (*n* = 86)		Controls (*n* = 58)	*p*-Value *
	Group A (*n* = 49)	Group B (*n* = 37)		
LTM (g)	27,757.4 ± 5758.9	26,801.3 ± 6078.5	28,436.8 ± 6115.1	0.56
LTM (%)	68.7 ± 8.9	69.9 ± 9.0	70.5 ± 8.0	0.52
FM (g)	12,002.8 ± 6748.1	10,874.7 ± 5781.3	11,082.7 ± 5395.3	0.47
FM (%)	28.8 ± 9.5	27.4 ± 9.5	27.1 ± 8.8	0.59
***Total-body-less-head***				
BMC (g)	1420.3 ± 290.9	1475.4 ± 475.3	1509.2 ± 354.1	0.86
BMD (g/cm^2^)	0.82 ± 0.08 ^#^	0.85 ± 0.11	0.85 ± 0.07	**0.04**
BMD Z-score	0.41 ± 0.91 ^†^	0.50 ± 0.77 ^†^	0.84 ± 0.63	**0.03**
BMD Z-score < −1.0 SD [n (%)]	4 (8.2) ^#^	0 (0)	0 (0)	**0.03**
***Lumbar-spine (L1–L4)***				
BMC (g)	28.7 ± 6.4 ^#^	32.1 ± 11.8	31.52 ± 9.05	**0.03**
Bone area (cm^2^)	37.5 ± 5.5	37.7 ± 7.1	37.8 ± 6.3	0.97
BMD (g/cm^2^)	0.76 ± 0.10 ^†^	0.83 ± 0.16	0.82 ± 0.11	**0.05**
BMD Z-score	−0.34 ± 0.84 ^‡^	−0.09 ± 0.77 ^#^	0.23 ± 0.71	**0.006**
BMD Z-score < −1.0 SD [*n* (%)]	11 (22.4) ^#^	5 (16.7)	4 (6.9)	**0.05**

LTM, lean tissue mass; FM, total body fat mass; BMC, bone mineral content; BMD, bone mineral density. Group A: children born very preterm (≤32 gestational weeks). Group B: children born moderately or late preterm (32^+1^ to 36^+6^ gestational weeks). Statistical significance is defined by *p*-value less than or equal to 0.05 and statistically significant results are shown in bold type; * *p*-values from analysis of variance (ANOVA) or Kruskal–Wallis test (H test), as appropriate, for continuous variables and from chi-square test for categorical variables. For pairwise subgroup comparisons, ^#^ *p* ≤ 0.05, ^†^ *p* ≤ 0.01, and ^‡^ *p* ≤ 0.001 in comparison with controls, after Bonferroni correction. Exact Bonferroni-adjusted *p*-values from posthoc pairwise subgroup comparisons are mentioned in the main text and in Table S3 (Supplementary Materials). Body regions assessed by Dual-energy X-ray absorptiometry (DXA) are presented in italics.

**Table 4:** Perinatal and neonatal characteristics associated with low BMD (Z-score ≤ −1.0 SD) in children born preterm.

Variable	Very Preterm-Born Children with Low BMD (*n* = 13)	Very Preterm-Born Children with Normal BMD (*n* = 36)	*p*-Value
Age (years)	11.0 ± 1.6	10.8 ± 1.0	0.80
Males (*n*)	8	16	0.29
Small for gestational age (SGA) [*n* (%)]	1 (7.7)	2 (5.6)	0.92
Maternal age at birth (years)	34.6 ± 5.9	33.6 ± 4.3	0.54
Maternal gestational hypertension [*n* (%)]	1 (7.7)	0 (0)	NA
Maternal preeclampsia [n (%)]	3 (23.1)	4 (11.1)	0.38
Maternal gestational diabetes [*n* (%)]	1 (7.7)	6 (16.7)	0.35
Maternal smoking during pregnancy [*n* (%)]	1 (7.7)	6 (16.7)	0.35
Antenatal corticosteroids [*n* (%)]	6 (46.2)	23 (63.9)	0.96
Cesarean delivery [*n* (%)]	12 (92.3)	32 (88.9)	0.73
Gestational age (weeks)	29.0 ± 2.5	29.0 ± 2.1	0.98
Birth weight (g)	1242.9 ± 386.2	1258.8 ± 347.1	0.89
RDS [*n* (%)]	10 (76.9)	30 (83.3)	0.33
Surfactant therapy [*n* (%)]	10 (76.9)	25 (69.4)	0.93
Mechanical ventilation [*n* (%)]	12 (92.3)	28 (77.8)	0.39
Duration of mechanical ventilation (days)	14.0 (1.0–38.5)	10.5 (2.3–29.0)	0.90
Duration of parenteral nutrition (days)	38.5 (12.5–48.5)	30.0 (17.5–37.5)	0.72
BPD [*n* (%)]	7 (53.8)	12 (33.3)	0.21
IVH [*n* (%)]	5 (38.5)	12 (33.3)	0.75
ROP [*n* (%)]	5 (38.5)	11 (30.6)	0.23
PDA [*n* (%)]	3 (23.1)	9 (25.0)	0.80
NEC [*n* (%)]	2 (15.4)	1 (2.8)	0.13
Weight (kg)	34.5 ± 6.4	44.2 ± 11.5	**0.01**
Weight Z-score	−0.38 ± 0.64	0.82 ± 0.90	**<0.001**
Height (cm)	144.8 ± 11.2	146.2 ± 9.6	0.67
Height Z-score	0.10 ± 0.93	0.49 ± 0.92	0.20
BMI (kg/m^2^)	16.5 ± 2.8	20.5 ± 3.7	**0.001**
BMI Z-score	−0.74 ± 1.38	0.84 ± 0.94	**0.002**
Waist circumference (cm)	65.1 ± 7.5	75.6 ± 10.3	**0.002**
Hip circumference (cm)	70.3 ± 7.3	81.8 ± 8.8	**0.004**
WHR	0.89 ± 0.06	0.93 ± 0.04	**0.05**
Tanner stage 1–2 [*n* (%)]	11 (84.6)	23 (63.9)	0.20
Previous fracture history [*n* (%)]	0 (0)	1 (2.8)	NA
Inadequate daily calcium intake [*n* (%)]	5 (38.5)	2 (5.6)	**<0.001**
Physical activity (hours per week)	5.0 (2.8–6.3)	5.0 (4.0–6.0)	0.94
Calcium (mg/dL)	9.84 ± 0.20	9.79 ± 0.22	0.48
Phosphorus (mg/dL)	4.60 ± 0.36	4.42 ± 0.42	0.17
Alkaline phosphatase (IU/L)	270.92 ± 69.66	275.78 ± 69.55	0.83
25(OH)D (ng/mL)	24.04 ± 8.23	22.55 ± 9.38	0.71
Osteocalcin (OC) (ng/mL)	26.56 ± 4.46	27.27 ± 5.64	0.81
Procollagen type I C-terminal propeptide (PICP) (ng/mL)	531.75 ± 322.98	457.00 ± 293.56	0.67
Insulin growth factor-1 (IGF-1) (ng/mL)	188.25 ± 95.39	352.45 ± 162.57	**0.04**
Serum tartrate-resistant acid phosphatase 5b (bone TRAP5b) (U/L)	7.20 ± 1.30	8.17 ± 2.19	0.32
Urinary calcium/creatinine (uCa/uCr)	0.37 (0.15–0.52)	0.26 (0.11–0.53)	0.62

SGA, small for gestational age; RDS, respiratory distress syndrome; BPD, bronchopulmonary dysplasia; IVH, intraventricular hemorrhage; ROP, retinopathy of prematurity; PDA, patent ductus arteriosus; NEC, necrotizing enterocolitis; BMI, body mass index; WHR, waist-to-hip ratio; 25(OH)D, 25-hydroxyvitamin D; NA, not applicable. Statistical significance is defined by *p*-value less than or equal to 0.05 and statistically significant results are shown in bold type.

## Data Availability

The data presented in this study are available upon reasonable request and should be made to Tania Siahanidou (siahan@med.uoa.gr). The data are not publicly available due to privacy.

## Supplementary Materials

The following supporting information can be downloaded at https://www.mdpi.com/article/10.3390/metabo15070463/s1, Table S1: *p*-values of pairwise subgroup comparisons regarding perinatal and neonatal characteristics in preterm-born children and in controls; Table S2: *p*-values of pairwise subgroup comparisons regarding anthropometric characteristics and metabolic bone profile in preterm-born children and in controls; Table S3: *p*-values of pairwise subgroup comparisons regarding body composition and bone densitometry findings in preterm-born children and in controls.
