# Supplementary material for: The Long-Term Impact of Preterm Birth on Metabolic Bone Profile and Bone Mineral Density in Childhood
Source: Metabolites. 2025 Jul 8;15(7):463. doi: 10.3390/metabo15070463 (PMC12299712; doi:10.3390/metabo15070463)
Supplement: Supplementary file 1 [file metabolites-15-00463-s001.zip › metabolites-3707583-supplementary.pdf]

Table S1. *p*-Values of pairwise subgroup comparisons regarding perinatal and neonatal characteristics in preterm-born children and in controls.

Variable	Group A vs. Controls (*p*-Value)*	Group A vs. Group B (*p*-Value)*	Group B vs. Controls (*p*-Value)*
Age (years)	0.59	0.93	0.29
Males (n)	0.33	0.25	0.14
Small for gestational age (SGA) [n (%)]	0.43	0.34	0.29
Maternal age at birth (years)	0.50	0.47	0.64
Maternal gestational hypertension [n (%)]	0.37	0.68	0.34
Maternal preeclampsia [n (%)]	**0.01**	0.62	**0.03**
Maternal gestational diabetes [n (%)]	0.60	0.24	0.30
Maternal smoking during pregnancy [n (%)]	**0.01**	0.23	**0.002**
Antenatal corticosteroids [n (%)]	**<0.001**	**0.02**	**<0.001**
Cesarean delivery [n (%)]	**<0.001**	0.29	**<0.001**
Gestational age (weeks)	**<0.001**	**<0.001**	**<0.001**
Birth weight (g)	**<0.001**	**<0.001**	**<0.001**
RDS [n (%)]	**<0.001**	**<0.001**	**<0.001**
Surfactant therapy [n (%)]	**<0.001**	**<0.001**	**<0.001**
Mechanical ventilation [n (%)]	**<0.001**	**<0.001**	**<0.001**
Duration of mechanical ventilation (days)	**<0.001**	**<0.001**	**<0.001**
Duration of parenteral nutrition (days)	**<0.001**	**0.02**	**0.02**
BPD [n (%)]	**<0.001**	**<0.001**	N/A
IVH [n (%)]	**<0.001**	**0.002**	**0.02**
ROP [n (%)]	**<0.001**	**0.002**	**0.01**
PDA [n (%)]	**<0.001**	**<0.001**	N/A
NEC [n (%)]	0.09	0.47	0.38

SGA: small for gestational age, RDS: respiratory distress syndrome, BPD: bronchopulmonary dysplasia, IVH: intraventricular hemorrhage, ROP: retinopathy of prematurity, PDA: patent ductus arteriosus, NEC: necrotizing enterocolitis. Group A: children born very preterm (≤32 gestational weeks); Group B: children born moderately or late preterm (32^{+1} to 36^{+6} gestational weeks). Statistical significance is defined by p-value less than or equal to 0.05 and statistically significant results are shown in bold type. *Bonferroni-adjusted p-values from pairwise subgroup comparisons using independent t-test or Mann-Whitney test (for continuous variables) and chi-square test (for categorical variables).

Table S2. *p*-Values of pairwise subgroup comparisons regarding anthropometric characteristics and metabolic bone profile in preterm-born children and in controls.

**Variable**	**Group A vs. Controls (*p*-Value)***	**Group A vs. Group B (*p*-Value)***	**Group B vs. Controls (*p*-Value)***
Weight (kg)	0.40	0.09	0.67
Weight z-score	0.78	0.17	0.22
Height (cm)	0.73	0.46	0.89
Height z-score	0.67	0.75	0.49
BMI (kg/m^2)	0.37	**0.03**	0.27
BMI z-score	0.83	0.18	0.26
Waist circumference (cm)	0.08	**0.001**	0.63
Hip circumference (cm)	0.92	0.62	0.74
WHR	**0.001**	**0.002**	0.33
Tanner stage 1-2 [n (%)]	0.06	0.46	0.06
Previous fracture history [n (%)]	0.29	0.62	0.06
Inadequate daily calcium intake [n (%)]	0.08	0.06	0.12
Physical activity (hours per week)	**0.005**	**0.002**	0.46
Calcium (mg/dL)	0.06	0.06	0.88
Phosphorus (mg/dL)	0.30	0.94	0.32
Alkaline phosphatase (IU/L)	**0.008**	**<0.001**	0.25
25(OH)D (ng/mL)	0.93	0.12	0.08
Osteocalcin (OC) (ng/mL)	**0.04**	**<0.001**	**0.002**
Procollagen type I C-terminal propeptide (PICP) (ng/mL)	**0.009**	**0.001**	**0.05**
Insulin growth factor-1 (IGF-1) (ng/mL)	0.32	0.53	0.63
Serum tartrate-resistant acid phosphatase 5b (bone TRAP5b) (U/L)	**0.01**	**<0.001**	**0.003**
Urinary calcium/creatinine (uCa/uCr)	0.30	0.30	0.56

BMI: body mass index, WHR: waist-to-hip ratio, 25(OH)D: 25-hydroxyvitamin D. Group A: children born very preterm (≤32 gestational weeks); Group B: children born moderately or late preterm (32^{+1} to 36^{+6} gestational weeks). Statistical significance is defined by p-value less than or equal to 0.05 and statistically significant results are shown in bold type. *Bonferroni-adjusted p-values from pairwise subgroup comparisons using independent t-test or Mann-Whitney test (for continuous variables) and chi-square test (for categorical variables).

Table S3. *p*-Values of pairwise subgroup comparisons regarding body composition and bone densitometry findings in preterm-born children and in controls.

Variable	Group A vs. Controls (*p*-Value)*	Group A vs. Group B (*p*-Value)*	Group B vs. Controls (*p*-Value)*
LTM (g)	0.56	0.34	0.32
LTM (%)	0.28	0.49	0.83
FM (g)	0.52	0.32	0.82
FM (%)	0.36	0.45	0.99
Total body less head			
BMC (g)	0.15	0.83	0.81
BMD (g/cm²)	**0.03**	0.38	0.91
BMD Z-score	**0.005**	0.86	**0.002**
BMD Z-score < -1.0 SD [n (%)]	**0.03**	0.52	0.11
Lumbar spine (L1-L4)			
BMC (g)	**0.05**	0.26	0.70
Bone area (cm²)	0.94	0.91	0.93
BMD (g/cm²)	**0.002**	0.09	0.82
BMD Z-score	**<0.001**	0.35	**0.05**
BMD Z-score < -1.0 SD [n (%)]	**0.02**	0.61	0.43

LTM: lean tissue mass, FM: total body fat mass, BMC: bone mineral content, BMD: bone mineral density. Group A: children born very preterm (≤32 gestational weeks); Group B: children born moderately or late preterm (32^{+1} to 36^{+6} gestational weeks). Statistical significance is defined by p-value less than or equal to 0.05 and statistically significant results are shown in bold type. *Bonferroni-adjusted p-values from pairwise subgroup comparisons using independent t-test or Mann-Whitney test (for continuous variables) and chi-square test (for categorical variables). Body regions assessed by Dual-energy X-ray absorptiometry (DXA) are presented in italics.
